# Connectivity Profile and Function of Uniquely Human Cortical Areas

**DOI:** 10.1523/JNEUROSCI.2017-24.2025

**Published:** 2025-03-17

**Authors:** Katherine L. Bryant, Julia Camilleri, Shaun Warrington, Guilherme Blazquez Freches, Stamatios N. Sotiropoulos, Saad Jbabdi, Simon Eickhoff, Rogier B. Mars

**Affiliations:** ^1^Wellcome Centre for Integrative Neuroimaging, Centre for Functional MRI of the Brain (FMRIB), Nuffield Department of Clinical Neurosciences, John Radcliffe Hospital, University of Oxford, Oxford OX3 9DU, United Kingdom; ^2^Institute for Language, Cognition and the Brain (ILCB), Aix-Marseille University, Marseille 13604, France; ^3^Institute of Neuroscience and Medicine: Brain and Behavior (INM-7), Research Center Jülich, Jülich 52428, Germany; ^4^Sir Peter Mansfield Imaging Centre, School of Medicine, University of Nottingham, Nottingham NG7 2QX, United Kingdom; ^5^Donders Institute for Brain, Cognition and Behaviour, Radboud University, Nijmegen 6500 HD, The Netherlands; ^6^National Institute for Health Research (NIHR) Nottingham Biomedical Research Centre, Queens Medical Centre, Nottingham NG1 5DU, United Kingdom; ^7^Institute of Systems Neuroscience, Medical Faculty, Heinrich Heine University Düsseldorf, Düsseldorf 40225, Germany

**Keywords:** comparative, connectivity, frontal, human uniqueness, social, temporal cortex

## Abstract

Determining the brain specializations unique to humans requires directly comparable anatomical information from other primates, especially our closest relatives. Human (*Homo sapiens*; m/f), chimpanzee (*Pan troglodytes*; f), and rhesus macaque (*Macaca mulatta*; m/f) white matter atlases were used to create connectivity blueprints, i.e., descriptions of the cortical gray matter in terms of the connectivity with homologous white matter tracts. This allowed a quantitative comparison of cortical organization across the species. We identified human-unique connectivity profiles concentrated in temporal and parietal cortices and hominid-unique organization in the prefrontal cortex. Functional decoding revealed human-unique hotspots correlated with language processing and social cognition. Overall, our results counter models that assign primacy to the prefrontal cortex for human uniqueness.

## Significance Statement

Understanding what makes the human brain unique requires direct comparisons with other primates, particularly our closest relatives. Using connectivity blueprints, we compared the cortical organization of the human brain to that of the macaque and, for the first time, the chimpanzee. This approach revealed human-specific connectivity patterns in the temporal and parietal lobes, regions linked to language and social cognition. These findings challenge traditional views that prioritize the prefrontal cortex in defining human cognitive uniqueness, emphasizing instead the importance of temporal and parietal cortical evolution in shaping our species’ abilities.

## Introduction

Our human behavioral repertoire enables us to spread across the globe into a much greater variety of niches than any other primate. Various behavioral innovations have alternatively been suggested to characterize our abilities, including our collaborative social abilities, tool use, the ability for mental time travel, and spoken language (Tomasello and Vaish, 2013; Healy, 2021; Suddendorf et al., 2022).

Understanding the basis of uniquely human behavior requires a comparison of our brain to that of our closest primate relatives. Such comparisons tend to focus on measures of size, highlighting that the human neocortex or cerebellum is expanded (Barton and Venditti, 2014), that certain areas are preferentially expanded (Donahue et al., 2018), or that the absolute number of neurons in the human brain outstrips that of other primates (Herculano-Houzel, 2012). None of these measures, however, provides a link to the behavior that the brain produces, and that, ultimately, is the likely target of selection. In contrast, work in neuroimaging has highlighted measures of brain organization at the level of areal connections that do have predictive value for the function of parts of the brain (Saygin et al., 2016; Mars et al., 2018a). Hence, the level of large-scale connections between brain areas is a more suitable level of between-species comparison of brain organization when one wants to understand the unique abilities of the human brain in the context of other primates.

Connectivity can now be studied at the whole-brain level using diffusion MRI and associated tractography algorithms, offering a new type of data for comparative and evolutionary neuroscience (Thiebaut de Schotten and Forkel, 2022). Recent work has created standardized protocols for reconstructing the major fiber pathways of the primate brain, creating white matter atlases of the human, developing human, and macaque monkey brains (Mars et al., 2018b; Warrington et al., 2022). These methods characterize the cortical areas of each species’ brain in terms of its connectivity with major white matter bundles, known to be homologous among primates. By describing all cortical areas of all brains in terms of connectivity to homologous tracts, we, in effect, place all the brains within a “common connectivity space.” This allows a quantitative comparison of brain organization across species (Mars et al., 2021). While previous studies focused on comparisons of the human brain with that of the most-often studied primate, the macaque, here we additionally exploit our recently developed comprehensive white matter atlases of the chimpanzee (Bryant et al., 2020), which allows us to directly compare humans with our closest relatives, as well as the macaque. To our knowledge, this is the first time the connectional organization of the entire cortex is compared between these species, although earlier comparisons of connections with a few specific tracts have been reported (Hecht et al., 2015; Sierpowska et al., 2022).

We described each point on the cortical surface of the human and chimpanzee brains as a vector of connectivity probabilities with 18 white matter fiber bundles that are homologous across species. We can then quantify which areas of the human brain diverge in terms of connectivity from those of the other species. Next, we assess how the connectivity profile of areas of divergence in humans differs from that of the closest match in the other species by identifying which connections are driving the observed differences in brain organization. Finally, we use meta-analytic data on functional brain activation to investigate the functional roles of divergent regions in the human brain, linking the anatomical differences between species’ brains to behavior.

## Materials and Methods

### Human data

Thirty human subjects (16 female, aged 22–35) were selected from the in vivo diffusion MRI data provided by the Human Connectome Project (HCP), WU-Minn Consortium (principal investigators: David Van Essen and Kamil Ugurbil; 1U54MH091657) funded by 16 NIH institutes and centers and the McDonnell Center for Systems Neuroscience at Washington University (Van Essen et al., 2013). Minimally preprocessed datasets from the Q2 public data release were used. Data acquisition and preprocessing methods have been previously described (Glasser et al., 2013; Sotiropoulos et al., 2013). Briefly, 1.25 mm isotropic resolution diffusion-weighted data were collected on a 3 T Siemens Skyra scanner with a slice-accelerated gradient echo EPI readout. *Q*-space sampling included three shells at *b *= 1,000, 2,000, and 3,000 s/mm^2^. Ninety diffusion encoding gradient directions and six *b* = 0 s were obtained twice for each shell, with the phase-encoding direction reversed. An MPRAGE sequence was used to acquire T1-weighted (T1w) images at 0.7 mm isotropic resolution and then aligned to diffusion space using the HCP minimal preprocessing pipeline (Glasser et al., 2013). Diffusion-weighted images were processed with FSL, using FMRIB's Diffusion Toolbox and bedpostX (Behrens et al., 2007). A high-resolution surface mesh (∼164,000 vertices per hemisphere) and a lower-resolution mesh (32,000 vertices per hemisphere) were generated using the PostFreeSurfer pipeline.

### Chimpanzee data

Chimpanzee (*Pan troglodytes*; *n* = 23, 26 ± 11 years, all female) MR scans were obtained from an archive hosted by the National Chimpanzee Brain Resource. Scans were acquired prior to the 2015 implementation of US Fish and Wildlife Service and National Institutes of Health regulations governing research with chimpanzees. All the scans reported here were collected as part of a grant to study aging in female primates, were completed by 2012, and have been used in previous studies (Autrey et al., 2014; Bryant et al., 2019, 2020). Chimpanzees were housed at the Emory National Primate Research Center (ENPRC), and all procedures were carried out in accordance with protocols approved by the ENPRC and the Emory University Institutional Animal Care and Use Committee (IACUC approval #YER-2001206).

Following standard ENPRC veterinary procedures, chimpanzee subjects were immobilized with ketamine injections (2–6 mg/kg, i.m.) and then anesthetized with an intravenous propofol drip (10 mg/kg/h) prior to scanning. Subjects remained sedated for the duration of the scans as well as the time required for transport between the scanner and their home cage. Primates were housed in a single cage for 6–12 h after scanning to recover from the effects of anesthesia before being returned to their home cage and cage mates. Veterinary and research staff evaluated the well-being of chimpanzees twice daily after the scan for possible postanesthesia distress.

MR scanning protocols and preprocessing for the chimpanzee dataset have been described in detail previously (Autrey et al., 2014). Briefly, anatomical and diffusion MR scans were acquired in vivo in a Siemens 3 T Trio scanner (Siemens Medical Systems). Diffusion-weighted MRI data were collected with a single-shot, pulsed-gradient spin-echo echo-planar imaging sequence. Parameters were as follows: 41 slices were scanned at a voxel size of 1.8 mm^3^, TR/TE of 5,900 ms/86 ms, and matrix size of 72 × 128. Two diffusion-weighted images were acquired for each of 60 diffusion directions (*b* = 1,000 s/mm^2^), each with one of the possible left–right phase-encoding directions and eight averages, allowing for correction of susceptibility-related distortion (Andersson et al., 2003). For each averaged diffusion-weighted image, six images without diffusion weighting (*b* = 0 s/mm^2^) were also acquired. High-resolution T1w and T2w images were acquired. Diffusion-weighted images were processed using FMRIB's Diffusion Toolbox and bedpostX (Behrens et al., 2007). Template generation for chimpanzees previously described in detail (Li et al., 2010) involved the PreFreeSurfer pipeline which was used to align the T1w and T2w volumes of 29 individual chimpanzees to native anterior commissure–posterior commissure space. Cortical surfaces and registrations to a population-specific chimpanzee template were generated using a modified version of the HCP minimal preprocessing pipeline (Glasser et al., 2013). The PostFreeSurfer pipeline was used to produce a high-resolution surface mesh (164,000 vertices) and a lower-resolution mesh (20,000 vertices).

### Macaque data

Eight postmortem macaque brain scans (*Macaca mulatta*, *n* = 8; six male; age range, 4–14 years) were acquired using a 7 T magnet with an Agilent DirectDrive console (Agilent Technologies). Acquisition and preprocessing have been detailed previously (Folloni et al., 2019). In brief, a 2D diffusion-weighted spin-echo protocol was implemented (DW-SEMS; TE/TR, 25 ms/10 s; matrix size, 128 × 128; resolution, 0.6 × 0.6 mm; number of slices, 128; slice thickness, 0.6 mm). Nine nondiffusion-weighted (*b* = 0 s/mm^2^) and 131 diffusion-weighted (*b* = 4,000 s/mm^2^) volumes were acquired with diffusion directions distributed over the whole sphere. The *b* = 0 images were averaged, and spatial signal inhomogeneities were restored. Ex vivo tissue usually has reduced diffusivity, necessitating larger *b*-values to achieve equivalent diffusion contrast to in vivo data; this was achieved here by increasing the diffusion sensitization from *b* = 1,000 to 4,000 s/mm^2^. Diffusion-weighted images were processed using the same method as chimpanzees, described above. The cortical surface of one macaque with high-quality structural MRI was reconstructed using a modified version of the HCP pipeline, nonlinearly registered to the other brains using FSL's FNIRT, warped to the other macaque brains, and transformed to F99 standard space (Van Essen, 2002).

### Between-species comparison based on white matter tracts

Eighteen major white matter bundles were reconstructed for all three species using probabilistic tractography (Behrens et al., 2007). A set of standardized masks previously developed for the human, chimpanzee, and macaque brains were used to reconstruct tracts based on objective anatomical landmarks that could be identified in all species. The logic behind this approach is that a set of seed, waypoint, stop, and exclusion masks are used to define the body of any white matter tract; the tractography algorithm is then free to reconstruct the rest of the bundle, including its gray matter termination points. In this way, we have something we can objectively define as homologous across the species (the body of the tract based on anatomical criteria) and something that varies across species and is the target of our investigation (the gray matter terminations; Mars et al., 2018b; Warrington et al., 2020).

All combinations of seed, waypoint, stop, and exclusion masks are described in detail in previous communications (Mars et al., 2018b; Warrington et al., 2020). The white matter tracts studied in the present study were the anterior commissure (AC); arcuate fascicle (AF); perigenual, dorsal, and temporal subdivisions of the cingulum bundle (CBP, CBD, and CBT, respectively); corticospinal tract (CST); frontal aslant (FA); forceps major (FMA); forceps minor (FMI); fornix (FX); inferior fronto-occipital fascicle (IFO); inferior longitudinal fascicle (ILF); middle longitudinal fascicle (MdLF); first, second, and third branches of the superior longitudinal fascicle (SLF1, SLF2, and SLF3, respectively); uncinate fascicle (UNC); and vertical occipital fascicle (VOF).

To assess the connectivity of each vertex of the cortical surface with each white matter fiber bundle, we created (surface) × (tract) matrices which we term “connectivity blueprints.” First, tractography is performed from each vertex of the cortical surface toward all voxels of the whole-brain white matter, creating a (brain) × (surface) matrix of connectivity. Then, each tract's tractogram, of the format (brain) × (tract), is premultiplied by the transposed (brain) × (surface) matrix, resulting in the (surface) × (tract) connectivity blueprint. The columns of this blueprint represent the surface projection of each tract, and the rows of the blueprint represent the connectivity profile of each vertex of the cortical surface. This method was first applied by Mars et al. (2018b) and is now implemented in FSL's XTRACT tool (Warrington et al., 2022; Assimopoulos et al., 2024).

Blueprints were averaged across subjects in each species to create a species-specific connectivity blueprint. Connectivity profiles can be compared across species by calculating the (vertex) × (vertex) Kullback–Leibler (KL) divergence between two common connectivity spaces. The best match of a vertex in one species is then found by finding the vertices with the lowest KL value (<2) in the other species. A spatial map of divergence of connectivity of one brain compared with another can be established by assigning to each vertex of the first brain the smallest KL value (minKL) across all vertices in the second brain.

### Functional decoding

To assess the functional roles of the areas of the human cortex that showed the greatest difference with the chimpanzee and the macaque, we used BrainMap, a publicly available meta-analytic database of functional activation studies (www.brainmap.org; Fox and Lancaster, 2002). BrainMap uses a structured standardized coding scheme to describe published human functional neuroimaging results. In particular, “behavioral domains” are categories and subcategories that aim to classify the cognitive functions likely to be isolated by any experimental contrast.

Functional decoding was done as follows. First, the cortex was divided into distinct regions according to the Glasser parcellation (Glasser et al., 2016). Each region was assigned the maximum within-region divergence score, i.e., the divergence value from the vertex that had the highest minKL value in the region. Second, we queried the BrainMap database in 2019 to assign the functional profile of these regions using forward inference (Eickhoff et al., 2011). Using forward inference, a cluster's functional profile is determined by identifying taxonomic labels for which the probability of finding activation in the respective cluster was significantly higher than the a priori chance (across the entire database) of finding activation in that particular cluster. Significance was established using a binomial test (*p* < 0.05, FDR corrected; Genovese et al., 2002). In other words, we tested whether the conditional probability of activation given a particular label [P(Activation|Task)] was higher than the baseline probability of activating the brain region in question per se [P(Activation)].

## Results

### Between-species comparison of connectivity blueprints

**Figure 1.**
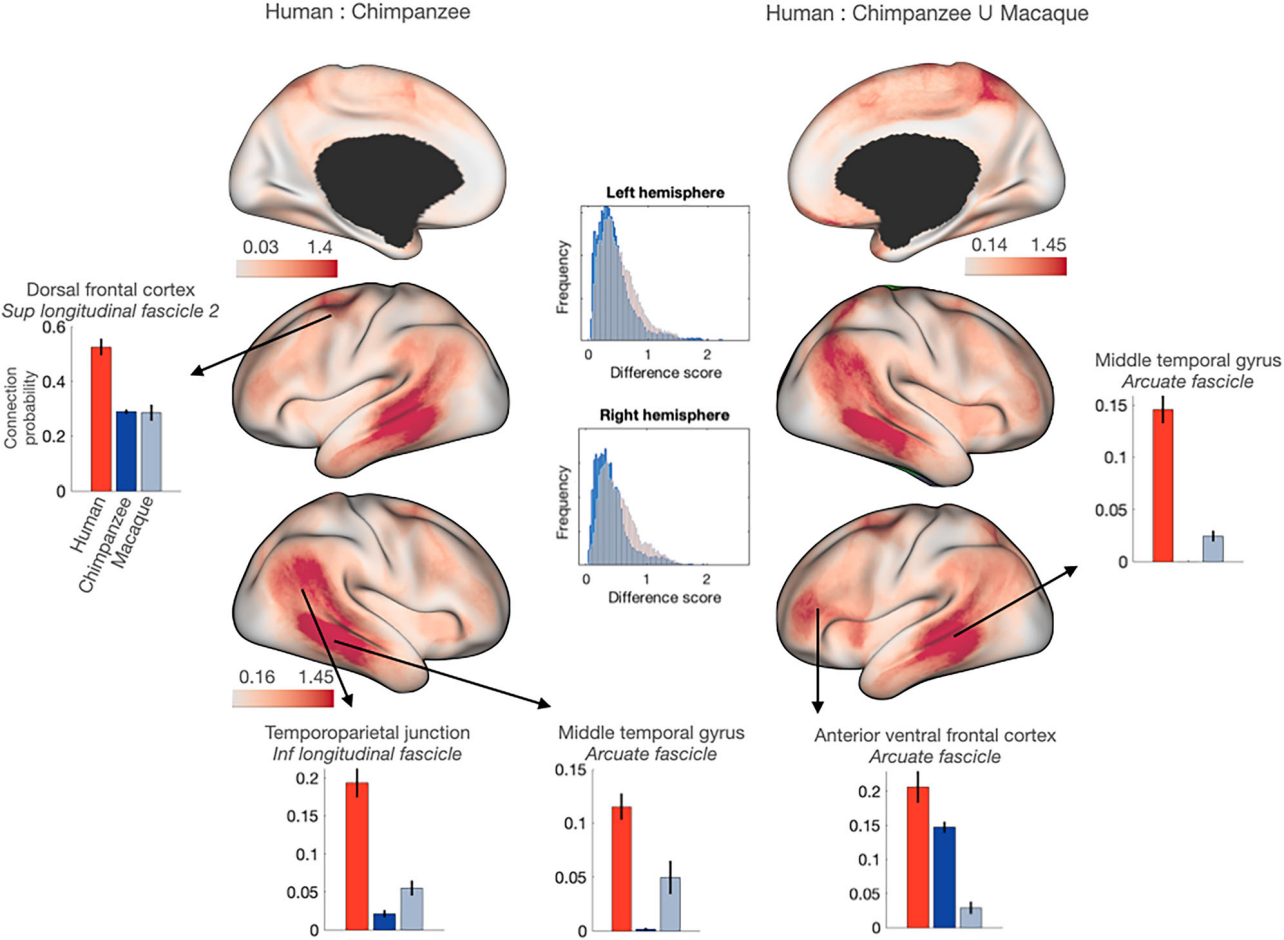
Mapping connectivity divergence between primates identifies multiple hotspots of human specialization. Here we show divergence maps of the human brain showing vertices with connectivity profiles that have a poor match in the chimpanzee (left) or in either the chimpanzee or the macaque (right). Bar graphs show the normalized connectivity (±SEM) of the selected vertex with a tract driving these differences in the human (red) and of its best-matching vertices in the chimpanzee (dark blue) and macaque (light blue). Tracts include SLF2 (superior longitudinal fascicle 2), ILF (inferior longitudinal fascicle), and AF (arcuate fascicle). Histograms in the center show the distribution of KL values comparing human and chimpanzee (blue) and human and macaque (red). The complete connectivity profile of each human vertex and its best matches are displayed in Extended Data Figure 1-1; the connectivity profile of anatomical homologs is displayed in Extended Data Figures 1-2–1-6.

For the human, chimpanzee, and macaque monkey brains, we established the connectivity of each part or vertex of the cortical surface with each of 18 white matter tracts that were determined in a homologous fashion in all three species. We term this (surface) × (tract) matrix the “connectivity blueprint.” The rows of this matrix describe the profile of connectivity of a given vertex of the cortical surface with each of the white matter tracts. The connectivity profile of any human vertex can be compared with that of each chimpanzee and macaque vertex by calculating the Kullback–Leibler (KL) divergence between connectivity profiles (Mars et al., 2018b). The best-matching vertex in the nonhuman species is the one with the minimum KL value. Overall spatial maps of divergence of the human brain from that of other species are then visualized by plotting the minimum KL value for each human vertex. When comparing the human with the chimpanzee brain, this shows large zones of divergence in the middle temporal lobe, temporoparietal cortex, and lateral frontal cortex with a particular hotspot in the dorsal frontal cortex (Fig. 1, left).

10.1523/JNEUROSCI.2017-24.2025.f1-1 Figure 1-1 Connectivity profile of areas of high between-species divergence in the human (red) and their best matching vertices in the chimpanzee (dark blue) and macaque (light blue). Download Figure 1-1, TIF file.

**Figure 2.**
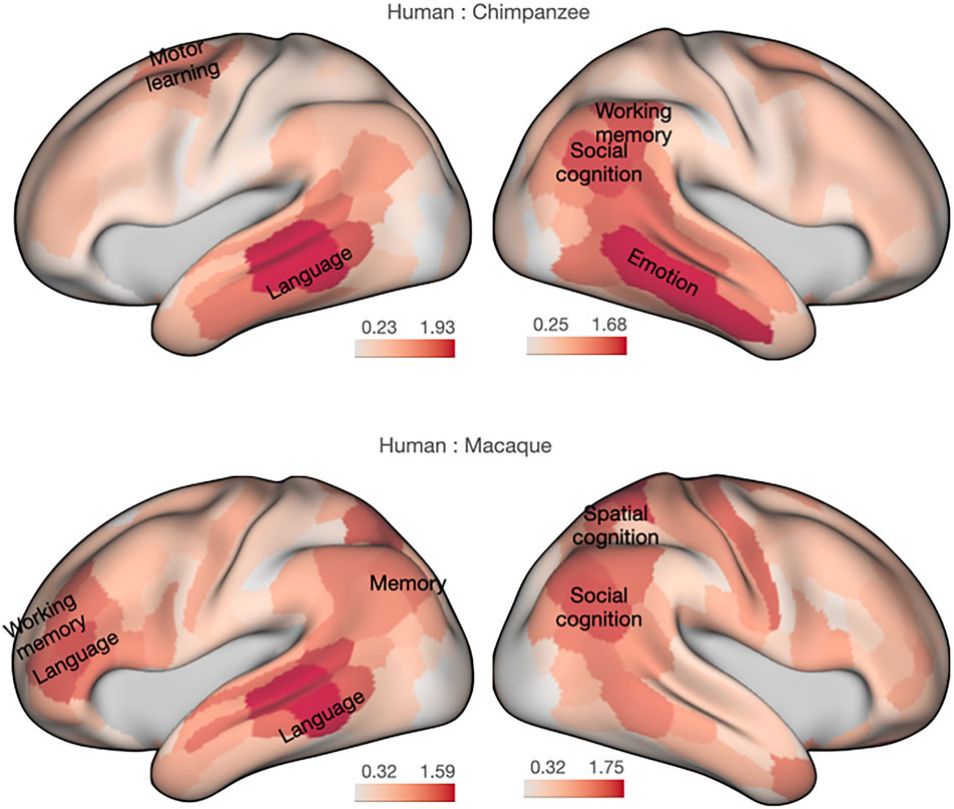
Decoding areas of high divergence highlight multiple behavioral domains. Functional activations that correlate most with areas of high KL divergence for the human and chimpanzee comparison (top) and the human and macaque comparison (****bottom). Color coding of areas according to the parcellation of Glasser et al. (2016) is done by assigning each area the divergence value of the most divergent vertex in that area. We note that the procedure of assigning a whole region with a single divergence value accentuates the spatial representation of this value and emphasizes that the actual vertex-wise presentation of Figure 1 presents the most spatially precise representation of the data. Full decoding of the areas is listed in the Extended Data Tables 2-1 and 2-2.

10.1523/JNEUROSCI.2017-24.2025.f1-2 Figure 1-2 Connectivity of human left dorsal frontal cortex with SLF2 (superior longitudinal fascicle 2, top right) and with all tracts (bottom right) in red and its chimpanzee and macaque homologs in dark and light blue, respectively. Download Figure 1-2, TIF file.

10.1523/JNEUROSCI.2017-24.2025.f1-3 Figure 1-3 Connectivity of human left anterior ventral frontal cortex with AF (arcuate fascicle, top right) and with all tracts (bottom right) in red and its chimpanzee and macaque homologs in dark and light blue, respectively. Download Figure 1-3, TIF file.

10.1523/JNEUROSCI.2017-24.2025.f1-4 Figure 1-4 Connectivity of human left middle temporal gyrus with AF (arcuate fascicle, top right) and with all tracts (bottom right) in red and its chimpanzee and macaque homologs in dark and light blue, respectively. Download Figure 1-4, TIF file.

10.1523/JNEUROSCI.2017-24.2025.f1-5 Figure 1-5 Connectivity of human right middle temporal gyrus with AF (arcuate fascicle, top right) and with all tracts (bottom right) in red and its chimpanzee and macaque homologs in dark and light blue, respectively. Download Figure 1-5, TIF file.

10.1523/JNEUROSCI.2017-24.2025.f1-6 Figure 1-6 Connectivity of human right temporoparietal junction (TPJ) areas with ILF (inferior longitudinal fascicle, top right) and with all tracts (bottom right) in red and its chimpanzee and macaque homologs in dark and light blue respectively. Download Figure 1-6, TIF file.

The divergence of the human brain from the chimpanzee brain can be compared with the divergence of the human brain from the macaque brain. The distribution of minimum KL values when comparing the human and the chimpanzee differs from that when comparing the human and the macaque (Kolmogorov–Smirnov test *p *< 0.001 for both hemispheres). Plotting the distribution of minimum KL values separately for the chimpanzee and the macaque indeed shows broader differences between the human and the macaque (Fig. 1, middle). Indeed, if we color each human vertex's divergence based on the species in which it was greatest, we see increases in divergence in the anterior ventral frontal cortex and posterior parietal cortex (Fig. 1, right).

The divergence between the human brain and both the chimpanzee and macaque brains was evident in the dorsal frontal cortex. The vertices of high divergence overlap with anterior area 6, the inferior 6–8 transition area, and the frontal eye fields (Glasser et al., 2016). The connectivity profile of this area is dominated by the frontal–parietal superior longitudinal fascicle, in particular, the second branch (SLF2; Thiebaut de Schotten et al., 2011; Fig. 1; see Extended Data Fig. 1-1 for full connectivity profiles). Using the common connectivity space, we can determine which vertices in the chimpanzee and the macaque have a connectivity profile that is the least different from that of the human. Extracting the connectivity of these vertices shows that even these do not show strong SLF2 connectivity (Fig. 1, Extended Data Fig. 1-2). We thus conclude that strong SLF2 connectivity in this part of the dorsal frontal cortex is driving the divergence in brain organization between the human and the other two primates.

Extensive differences between the human and nonhuman brains were found in the ventral frontal cortex and middle temporal gyrus. Both these hotspots of divergence were driven by more extensive connectivity of the arcuate fascicle (AF) in humans (Fig. 1). Such AF connectivity in the human brain has been shown before (Rilling et al., 2008; Sierpowska et al., 2022), but the comparison of the human with the chimpanzee, on the one hand, and the chimpanzee and macaque, on the other, shows a dissociation between the frontal and temporal cortices. While the best-matching vertices for the middle temporal cortex showed a lack of innervation of the AF in both chimpanzees and macaques, the best-matching vertices to the anteroventral frontal cortex show some AF in the chimpanzee, but none in the macaque. This suggests a scenario where the extension of the AF occurred gradually, with frontal expansions occurring in the ape lineage, preceding temporal expansions into the middle temporal cortex in the human lineage.

On the medial wall, we noticed a hotspot of divergence in medial parietal area 7. This divergence seems mostly driven by small changes in multiple tracts (Extended Data Fig. 1-1), rather than a clear elaboration of a single tract, as is the case for some of the divergent areas discussed above. However, the strongest connection of this area, SLF1, does seem more focal in the human than in the best-matching vertices in the other two species.

### Functional decoding of divergent regions

Next, we turned to a database of functional neuroimaging studies (brainmap.org; Fox et al., 2005) to assess the functional role of these regions. We assessed if, for a given behavioral domain, the probability of finding activation of a region was significantly higher than the a priori chance, so-called forward inference. This approach allows functional characterization of the areas we identified as structurally divergent from other primate brains (Fig. 2; Extended Data Tables 2-1, 2-2).

10.1523/JNEUROSCI.2017-24.2025.t2-1 Table 2-1 Download Table 2-1, DOCX file.

10.1523/JNEUROSCI.2017-24.2025.t2-2 Table 2-2 Download Table 2-2, DOCX file.

It is important to point out that the specificity of the decoding results can only be as good as the taxonomy of the BrainMap database. Thus, our results should not be taken such that any behavioral domain associated with an area constitutes the unique role of that area. Rather, the behavioral domain indicates the involvement of the area but does not claim the brain region is limited to that domain. We provide two tables showing the functional decoding of regions based on high divergence between the human and the chimpanzee (Extended Data ”Table 2-1) and between the human and the macaque monkey (Extended Data Table 2-2). Behavioral domains for significant decoding and likelihood ratios are reported. Regions are labeled according to the atlas of Glasser et al. (2016).

For the three dorsal frontal regions mentioned above, the behavioral domains most likely to activate them include spatial cognition, working memory, and reasoning. Some of these regions have previously been identified as part of the so-called multiple demand network (Assem et al., 2020), a network of mostly parietal and frontal regions that consistently activate for a range of high-level cognitive tasks. Although homologs of this network exist in the macaque, recent comparative work shows that the connections between these regions are much more extensive in the human (Karadachka et al., 2023). It has been suggested that human domain-general knowledge has a precursor in parietal–frontal network originally evolved for visuomotor control in early primates (Genovesio et al., 2014). The current results extend this finding to our nearest animal relative and directly link anatomical differences to functional domains associated with the multiple demand network.

Consistent with the role of the AF in human language, functional decoding of both the middle temporal and ventral frontal cortices in the left hemisphere yielded the behavioral domain “language” prominently. However, it was clear that the AF extension, especially in the temporal cortex, was bilateral. Decoding of the right middle temporal cortex yielded the domain “emotion.” Although the function of right temporal association cortices is yet not well-characterized in the fMRI literature, lesion studies suggest they play a role in nonverbal semantic social cognition (Binney et al., 2012). Importantly, these results speak against a language-only interpretation of AF extensions in the ape and human brains.

A prominent zone of divergence between the human brain and that of both the chimpanzee and macaque was in the posterior superior temporal cortex and inferior parietal lobule, together often referred to as the temporoparietal junction area (TPJ). This effect was particularly prominent in the right hemisphere. The right posterior TPJ especially has often been associated with the human ability to entertain others’ belief states, so-called mentalizing or theory of mind (Schurz et al., 2017). The hotspot of divergence overlaps with this area, and functional decoding indeed shows “social cognition” as its most significant behavioral domain. The human posterior TPJ shows strong connectivity to the inferior longitudinal fascicle (ILF), which is not present in the other two species (Extended Data Fig. 1-6). The ILF is part of the ventral visual pathway but extends into the parietal cortex in anthropoid primates (Roumazeilles et al., 2022). It is thought that the ILF has expanded in great apes and that the dorsal component has a role in social cognition, allowing some of the temporal cortex machinery for visual processing to be adapted for social information processing (Pitcher and Ungerleider, 2020; Roumazeilles et al., 2020). The current results connect these two findings of TPJ's role in social cognition and ILF's prominent expansion by showing that the TPJ is innervated by the ILF in the human.

### Comparison of connectivity profiles across species based on a priori homologs

It is important to note that the above analyses select those vertices in the chimpanzee and macaque brains that have the least divergent connectivity profile with the chosen vertex in the human brain, independent of their location. This allows an unbiased assessment of divergence across the different species’ brains. As has been shown previously, this analysis is capable of identifying homologous regions that are known to have similar connectivity profiles across species (Mars et al., 2018b) while not relying on priors. It is therefore more principled than comparing known homologs across species. For completion, however, we also present comparisons of the connectivity profiles of human areas with those of known homologs in the chimpanzee and macaque for all areas in Figure 1.

The left dorsal prefrontal region overlaps with anterior area 6, the inferior 6–8 transition area, and the frontal eye fields (Glasser et al., 2016). In humans, this area has much stronger connectivity to SLF2, compared with its best-matching chimpanzee and macaque counterparts. We extracted the connectivity profiles of area FB in the chimpanzee (Bailey et al., 1950), which has been suggested to contain the frontal eye fields (Percheron et al., 2015), and macaque FEF (Petrides, 2005). As with the best-matching vertices, the human has much stronger SLF2 connectivity in this territory than the other species (Extended Data Fig. 1-2).

The human anterior ventral frontal cortex received innervations of the arcuate fascicle (AF), which was evident to a lesser extent in the chimpanzee and absent in the macaque. The human area of maximum divergence overlaps with the area IFSa of Glasser et al. (2016) and the area IFS of Neubert et al. (2014). The homolog of this area in the chimpanzee is difficult to establish. We extracted the connectivity profile of a vertex in area FCBm (Bailey et al., 1950) in the chimpanzee and on the posterior bank of the inferior branch of the arcuate sulcus in the macaque. In both cases, these locations are, if anything, quite posterior and therefore more likely to detect AF connectivity than human IFS. Nevertheless, the pattern of most AF connectivity in the human, less in the chimpanzee, and very little in the macaque was replicated (Extended Data Fig. 1-3).

The human middle temporal gyrus shows strong AF connectivity, which is much lower even in the best-matching areas in the other two species. When extracting the connectivity profile of the middle temporal gyrus in the chimpanzee and macaque, this pattern of relatively reduced AF in the nonhuman primates is even stronger (Extended Data Figs. 1-4, 1-5).

The right temporoparietal junction (TPJ) area in the human brain shows strong innervation of the ILF, which is not seen in the best-matching vertices in the chimpanzee and macaque. The homolog of TPJ is difficult to establish. Although the area overlaps with area PGi of Glasser et al. (2016), it is uncertain whether it is homologous to area PG in the macaque (Pandya and Seltzer, 1982). Mars et al. (2012) identified two subregions of TPJ, which they labeled TPJp and TPJa, the posterior of which shows strong activation in social cognition tasks, as found in our decoding analysis. Connectivity profiles of regions in the macaque inferior parietal lobule do not show a prominent ILF, but rather the IFO and MdLF. In addition, the small macaque inferior parietal lobule shows strong connectivity with the AF, which does not extend ventrally as it does in the human, as discussed above (Extended Data Fig. 1-6).

## Discussion

Comparing brain organization across species typically involves detailed analysis of small parts of the brain using measures such as cytoarchitecture or transcriptomics, on the one hand, or comparisons of large subdivisions using global measures such as relative brain size, on the other. In contrast, here we compared the organization of the human cortex directly with that of two other species at a level of direct relevance to function: connectivity. We exploit the availability of white matter atlases created using diffusion MRI to provide a detailed comparison of cortical organization between the human brain and that of one of its closest relatives, the chimpanzee, and the most often studied nonhuman primate, the macaque monkey. We demonstrate the uniquely human organization of large parts of the association cortex and relate them for the first time to the behavioral domains in which they show functional activation.

Although most debates regarding what might be special about the human brain focus on the prefrontal cortex (Barton and Venditti, 2013; Donahue et al., 2018), the current results demonstrate that major areas of difference between the human, chimpanzee, and macaque are in other parts of the association cortex. The most different region is in the middle temporal gyrus. This region was previously identified in our human–macaque comparisons (Mars et al., 2018b), and the current results extend this result to the human–chimpanzee comparison. This change is primarily driven by the extension of the arcuate fascicle. The arcuate expansion has been identified as a hallmark of human language (Rilling et al., 2008; Roelofs, 2014), but a focus solely on language might be a too narrow interpretation of this major between-species difference. For instance, the arcuate expansion is bilateral, and, although the right temporal cortex also has some language functions, our functional decoding shows its involvement in other functions as well. Moreover, the arcuate extension is partly driven by the short parietal–temporal aspect of the arcuate (Sierpowska et al., 2022) integrating information processing between the dorsal and ventral cortical pathways.

An important difference between the human–chimpanzee and the human–macaque comparisons is in the ventral frontal cortex. Although the cortical territory termed “Broca's area” has been associated with uniquely human organization and function, the picture of the precise pattern of evolutionary change is only now starting to become clear. When comparing the human to the chimpanzee, there is no clear hotspot of change in the ventral prefrontal cortex, whereas this is clear in the human–macaque comparison. This result extends earlier demonstrations of a difference in both areas 44 and 45 between the adult human and adult macaque brain, but only in area 44 between the adult macaque and the human infant (Warrington et al., 2022). Another prominent frontal cortex difference between the human and both nonhuman primates was in the strength of parietal–frontal connections. Some of these differences had been identified in human–macaque comparisons but are now shown to be unique to the human lineage.

The between-species differences in the temporal and temporoparietal cortex are not solely driven by the arcuate. It had previously been established that the temporal longitudinal white matter pathways are more extensive and show more complex subdivisions in apes than in monkeys (Roumazeilles et al., 2020). Here, we demonstrate that the inferior longitudinal fascicle reaches part of the so-called temporoparietal junction area (TPJ) in the human. This area has previously been shown to share some anatomical and functional properties with face-sensitive areas in the macaque middle superior temporal sulcus (Mars et al., 2013; Roumazeilles et al., 2021), but human TPJ seems to process the more complex information associated with human social cognition, by entertaining either others’ belief states (Koster-Hale et al., 2017) or the difference between one's own and other's knowledge (Kolling et al., 2021).

Differences between the human and nonhuman primates are less prominent on the medial wall, but the medial parietal cortex does show a hotpot of divergence between species. This dovetails with earlier reports comparing humans and macaques (Mars et al., 2018b). Precuneus has previously been identified as a region of expansion in the brain of modern humans based on fossil endocasts (Bruner, 2018). Here, we show that such changes are accompanied by changes in connectivity profile, although it is unknown whether the two types of changes coincided.

Our approach of using white matter tracts as a common space in which to describe the brain organization of the three species contrasts with that of a direct spatial registration of the brains based on sulcal morphology (Chaplin et al., 2013; Vickery et al., 2024). There are two reasons the common space approach is beneficial. First, the homology of sulci across the human, chimpanzee, and macaque brains is far from established. Major longitudinal sulci such as the macaque principal sulcus may not be homologous to any of the frontal sulci of the great apes (Petrides, 2005), and the pattern of smaller sulci is more complex in the human brain (Hathaway et al., 2024). Secondly, while sulcal-based registration might identify regional expansion and even relocation of certain cortical areas (Hill et al., 2010), these results do not speak to the different possible scenarios of evolutionary change that can accompany such changes, including whether a region has simply expanded or also changed its profile of connectivity with the rest of the brain (Eichert et al., 2020). In the latter case, the interaction of the region with other parts of the brain has changed, which likely results in different functional roles. Indeed, changes in the connectivity of cortical areas have been proposed to be a prominent way in which brain organization changes throughout evolution (Krubitzer and Kaas, 2005).

Although our approach addresses problems of differences in brain size and morphology when comparing different species’ brains, as with any method, it has some limitations that should be kept in mind when interpreting the results. Our definition of common tracts relies on the correct placement of seed, waypoint, stop, and exclusion masks for the tractography recipe of each tract. Our approach has been to define masks based on explicit anatomical landmarks that can be recognized easily across species. Previous work has validated these recipes compared with known tracts in the human and the macaque (Mars et al., 2018b; Warrington et al., 2020) and definitions of new species are created in as similar a way as possible. But we acknowledge that the tractography masks are the basis of the comparisons. The masks defined for the chimpanzee and their comparisons to the human and macaque have been the topic of a previous communication (Bryant et al., 2020). All recipes used in this approach can be found on the website of the XTRACT tool; the modular organization of XTRACT means that researchers can easily substitute their own recipes and study the effects on the between-species comparisons.

Due to the limited availability of data from the chimpanzee, our sample only consisted of female subjects. Similarly, our age range is limited to young adults for all species. Although to our knowledge differences in connectivity across sexes are limited to white matter volume and the strength of particular connections rather than the presence or absence of particular fiber bundles (Gong et al., 2011), subtle differences in connectivity across sexes and how these differences manifest themselves across species are important avenues in research. Translational neuroscience has long been biased by inclusion of mostly single-sex data, while it is now known that sex differences occur even in rodent brains (Guma et al., 2024). The connectivity blueprint method has been used to compare young adult and infant humans (Warrington et al., 2022), and developmental changes in other species are the topic of ongoing research, where the data are available. However, for the current study, the single time point and sex bias in the data are a limitation of the scope.

Although the comparison of the organization of the entire neocortex of the human to two other species of primate is unique, future work will strengthen and extend our results by inclusion of more species and direct comparisons across them. The current manuscript has focused on the human as the reference species, but a full understanding of primate phylogeny necessitates comparisons that are less human-centric. Using the same protocols as those used in the present study, partial white matter atlases for other primate species are now available (Bryant et al., 2021, 2023), and work to extend these to include the same range of tracts as the present study is ongoing. Moreover, data-driven methods for identification of white matter tracts have also shown promise in comparative studies (Mars et al., 2019).

Overall, our results thus argue against a single explanatory factor or evolutionary event driving the uniquely human behavioral repertoire. While current theories on human brain uniqueness focus on changes to prefrontal areas, our findings support a two-step evolutionary process, in which changes in prefrontal cortex organization emerge prior to changes in temporal areas. Unlike global connectivity or gross anatomical approaches, anatomically informed comparative connectivity makes it possible to reveal major changes in multiple association fiber systems underlying a variety of cognitive functions that have changed in a stepwise manner in the great ape and human lineages.
