# Supplementary figures and images for: Connectivity Profile and Function of Uniquely Human Cortical Areas
Source: J Neurosci. 2025 Mar 17;45(15):e2017242025. doi: 10.1523/JNEUROSCI.2017-24.2025 (PMC11984073; doi:10.1523/JNEUROSCI.2017-24.2025)

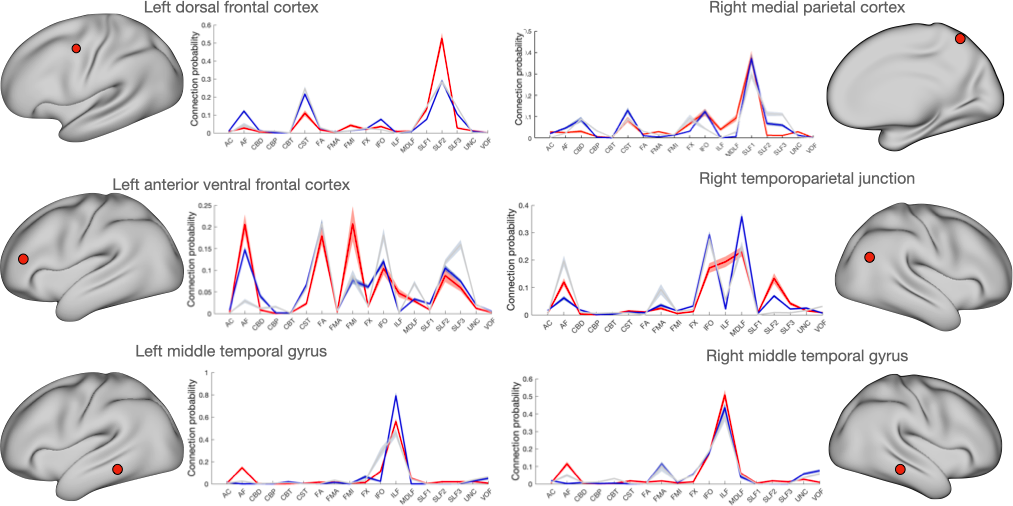

Supplement: Figure 1-1 — Connectivity profile of areas of high between-species divergence in the human (red) and their best matching vertices in the chimpanzee (dark blue) and macaque (light blue). Download Figure 1-1, TIF file. [file jneuro-45-e2017242025-s001.tif]

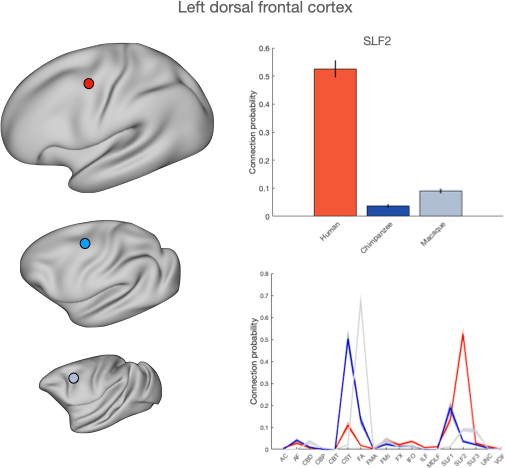

Supplement: Figure 1-2 — Connectivity of human left dorsal frontal cortex with SLF2 (superior longitudinal fascicle 2, top right) and with all tracts (bottom right) in red and its chimpanzee and macaque homologs in dark and light blue, respectively. Download Figure 1-2, TIF file. [file jneuro-45-e2017242025-s002.tif]

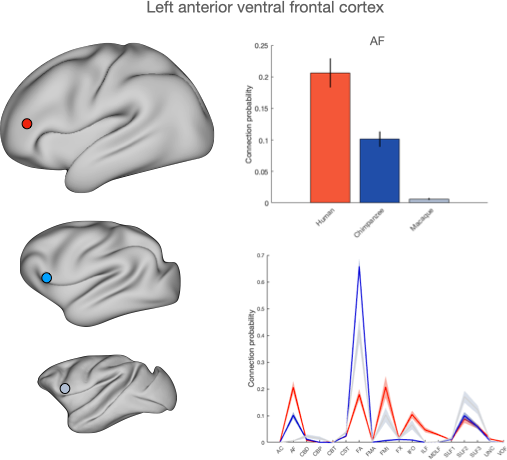

Supplement: Figure 1-3 — Connectivity of human left anterior ventral frontal cortex with AF (arcuate fascicle, top right) and with all tracts (bottom right) in red and its chimpanzee and macaque homologs in dark and light blue, respectively. Download Figure 1-3, TIF file. [file jneuro-45-e2017242025-s003.tif]

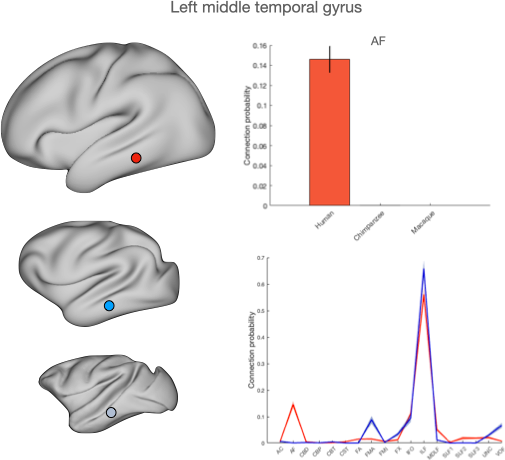

Supplement: Figure 1-4 — Connectivity of human left middle temporal gyrus with AF (arcuate fascicle, top right) and with all tracts (bottom right) in red and its chimpanzee and macaque homologs in dark and light blue, respectively. Download Figure 1-4, TIF file. [file jneuro-45-e2017242025-s004.tif]

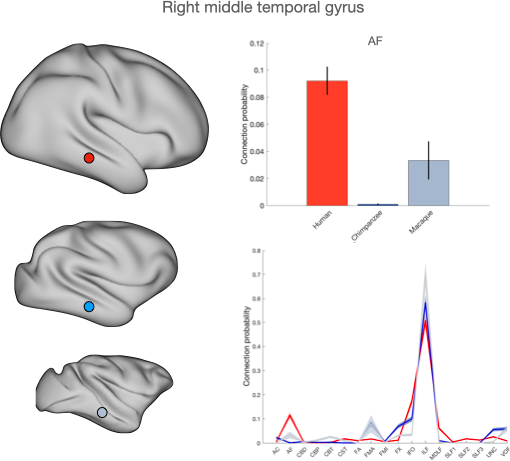

Supplement: Figure 1-5 — Connectivity of human right middle temporal gyrus with AF (arcuate fascicle, top right) and with all tracts (bottom right) in red and its chimpanzee and macaque homologs in dark and light blue, respectively. Download Figure 1-5, TIF file. [file jneuro-45-e2017242025-s005.tif]

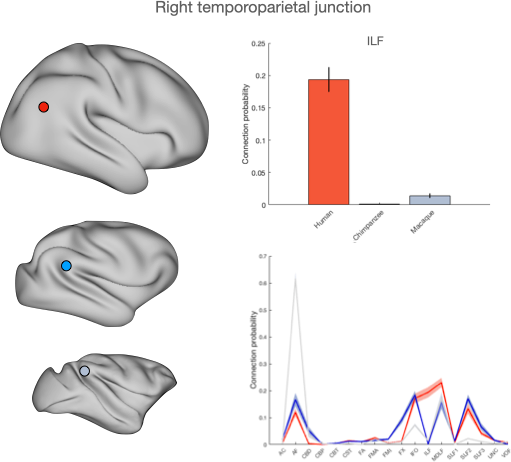

Supplement: Figure 1-6 — Connectivity of human right temporoparietal junction (TPJ) areas with ILF (inferior longitudinal fascicle, top right) and with all tracts (bottom right) in red and its chimpanzee and macaque homologs in dark and light blue respectively. Download Figure 1-6, TIF file. [file jneuro-45-e2017242025-s006.tif]
