# Supplementary material for: Connectivity Profile and Function of Uniquely Human Cortical Areas
Source: J Neurosci. 2025 Mar 17;45(15):e2017242025. doi: 10.1523/JNEUROSCI.2017-24.2025 (PMC11984073; doi:10.1523/JNEUROSCI.2017-24.2025)
Supplement: Table 2-1 — Download Table 2-1, DOCX file. [file jneuro-45-e2017242025-s007.docx]

| Human : chimpanzee | |  |  |  |  |  |  |  |
| --- | --- | --- | --- | --- | --- | --- | --- | --- |
|  |  |  |  |  |  |  |  |  |
| LEFT HEMISPHERE | |  |  |  | **RIGHT HEMISPHERE** | |  |  |
|  |  |  |  |  |  |  |  |  |
| Frontal |  |  |  |  | **Temporal** |  |  |  |
|  | 6ma | Action.Imagination | 3.41 |  |  | TE1p | No significant effects | 3.67 |
|  | i6-8 | No significant effects |  |  |  | PHT | Emotion.Negative.Disgust | 3.74 |
|  | 6a | Cognition.Reasoning | 1.4 |  |  | TPOJ2 | Action.Observation | 4.34 |
|  |  | Cognition.Memory.Working | 1.77 |  |  |  | Emotion.Negative.Disgust | 3.16 |
|  |  | Action.Execution | 1.82 |  |  | TPOJ3 | Cognition.Social Cognition | 3.67 |
|  |  | Perception.Vision.Shape | 1.96 |  |  |  | Perception.Vision.Shape | 4.62 |
|  |  | Action.Observation | 2.15 |  |  |  | Cognition.Spatial | 2.61 |
|  |  | Cognition.Spatial | 2.24 |  |  | TPOJ1 | Cognition.Social Cognition | 2.61 |
|  |  | Perception.Vision.Motion | 2.59 |  |  |  | Perception.Audition | 1.94 |
|  |  | Action.Imagination | 2.82 |  |  | TE1a | Cognition.Memory.Explicit | 2.35 |
|  |  | Action.Motor Learning | 5.3 |  |  |  | Emotion.Negative | 3.83 |
|  | FEF | Cognition.Spatial | 2.24 |  |  |  | Cognition.Social Cognition | 3.85 |
|  |  | Action.Execution | 2.52 |  |  |  | Perception.Audition | 4.99 |
|  |  | Action.Imagination | 2.89 |  |  |  | Cognition.Language | 5.98 |
|  |  | Perception.Vision.Motion | 3.44 |  |  |  | Emotion.Valence | 1.94 |
|  | 6d | Action.Imagination | 2.89 |  |  | STSdp | Cognition.Language.Semantics | 2.57 |
|  |  | Action.Execution | 4.27 |  |  |  | Cognition.Language.Speech | 3.71 |
|  |  | Action.Motor Learning | 6.43 |  |  |  | Emotion.Positive.Happiness | 3.79 |
| Temporal |  |  |  |  |  |  | Perception.Audition | 3.34 |
|  | STSva | Cognition.Memory.Explicit | 1.98 |  |  | STV | Cognition.Social Cognition | 2.42 |
|  |  | Cognition.Language.Semantics | 2.36 |  |  | PHT | Cognition.Spatial | 2.88 |
|  |  | Cognition.Social Cognition | 4.32 |  |  |  | Perception.Vision.Shape | 3.3 |
|  |  | Emotion.Valence | 5.34 |  |  |  | Interoception.Secuality | 4.44 |
|  |  | Cognition.Language | 5.58 |  |  |  | Action.Observation | 2.41 |
|  | A5 | Cognition.Music | 1.87 |  |  | FST | Perception.Vision | 2.66 |
|  |  | Cognition.Language | 2.78 |  |  |  | Emotion.Negative | 3.76 |
|  |  | Action.Execution.Speech | 2.96 |  |  |  | Perception.Vision.Motion | 3.94 |
|  |  | Cognition.Language.Speech | 3.31 |  |  |  | Action.Observation |  |
|  |  | Cognition.Language.Phonology | 3.76 |  | **Parietal** |  |  | 2.6 |
|  |  | Perception.Audition | 4.26 |  |  | PGs | Cognition.Social Cognition |  |
|  |  | Action.Motor Learning | 4.47 |  |  |  |  |  |
|  | STSdp | Cognition.Language.Semantics | 1.73 |  |  |  |  | 1.51 |
|  |  | Cognition.Language.Speech | 2.11 |  |  | PFm | Cognition.Reasoning | 1.86 |
|  |  | Cognition.Social Cognition | 2.15 |  |  |  | Perception.Somesthesis.Pain | 1.87 |
|  |  | Perception.Audition | 2.6 |  |  |  | Cognition.Memory.Working |  |
|  |  | Cognition.Language | 3.95 |  |  |  |  |  |
|  | TE1m | Cognition.Language.Semantics | 2.31 |  |  |  |  |  |
|  | TE1p | Cognition.Language.Speech | 2.34 |  |  |  |  |  |
|  |  | Cognition.Language.Orthography | 2.41 |  |  |  |  |  |
|  |  | Cognition.Language.Semantics | 2.48 |  |  |  |  |  |
|  |  | Cognition.Language.Phonology | 3.36 |  |  |  |  |  |
|  | PHT | Cognition.Language.Semantics | 1.82 |  |  |  |  |  |
|  |  | Introception.Sexuality | 2.92 |  |  |  |  |  |
|  |  | Action.Observation | 4.46 |  |  |  |  |  |
|  | TPOJ1 | Cognition.Language.Semantics | 1.94 |  |  |  |  |  |
|  |  | Cognition.Language.Speech | 2.06 |  |  |  |  |  |
|  |  | Perception.Audition | 2.53 |  |  |  |  |  |
|  |  | Emotion.Valence | 3.84 |  |  |  |  |  |
|  | STV | Cognition.Social Cognition | 2.4 |  |  |  |  |  |
|  |  | Cognition.Music | 2.46 |  |  |  |  |  |
|  |  | Emotion.Valence | 3.96 |  |  |  |  |  |
|  | TE1a | Cognition.Language.Semantics | 1.92 |  |  |  |  |  |
|  |  | Cognition.Language.Phonology | 2.86 |  |  |  |  |  |
|  |  | Cognition.Social Cognition | 4.63 |  |  |  |  |  |
|  |  | Cognition.Memory | 4.87 |  |  |  |  |  |
|  |  | Cognition.Language | 5.83 |  |  |  |  |  |
|  | TE2a | No significant effects |  |  |  |  |  |  |
| Parietal |  |  |  |  |  |  |  |  |
|  | PFt | Cognition.Language.Semantics | 2.39 |  |  |  |  |  |
|  |  | Cognition.Language.Phonology | 3 |  |  |  |  |  |
|  | PGi | Cognition.Memory.Explicit | 1.7 |  |  |  |  |  |
|  |  | Cognition.Social Cognition | 4.48 |  |  |  |  |  |
