# Supplementary material for: Connectivity Profile and Function of Uniquely Human Cortical Areas
Source: J Neurosci. 2025 Mar 17;45(15):e2017242025. doi: 10.1523/JNEUROSCI.2017-24.2025 (PMC11984073; doi:10.1523/JNEUROSCI.2017-24.2025)
Supplement: Table 2-2 — Download Table 2-2, DOCX file. [file jneuro-45-e2017242025-s008.docx]

| Human : Macaque | |  |  |  |  |  |  |  |
| --- | --- | --- | --- | --- | --- | --- | --- | --- |
|  |  |  |  |  |  |  |  |  |
| LEFT HEMISPHERE | |  |  |  | **RIGHT HEMISPHERE** | |  |  |
|  |  |  |  |  |  |  |  |  |
| Frontal |  |  |  |  | **Frontal** |  |  |  |
|  | p47r | No significant effects |  |  |  | 4 | Action.Execution | 3.17 |
|  |  |  |  |  |  |  | Action.Execution.Speech | 3.63 |
|  | IFSa | Cognition.Language.Semantics | 2.33 |  |  |  | Action.Motor Learning | 3.71 |
|  |  | Cognition.Language.Orthography | 2.47 |  |  |  | Interoception | 3.73 |
|  |  | Cognition.Language.Syntax | 3.01 |  |  | 6mp | Action.Execution | 2.51 |
|  |  | Cognition.Language.Phonology | 3.21 |  |  | OFC | Emotion.Positive | 4.57 |
|  |  |  |  |  |  | 10v | Cognition.Reasoning | 2.29 |
|  | Area 45 | Cognition.Memory.Explicit | 1.63 |  |  |  | Emotion.Positive.Reward/Gain | 3.17 |
|  |  | Emotion.Negative | 1.86 |  | **Temporal** |  |  |  |
|  |  | Cognition.Language.Speech | 1.96 |  |  | TGd | Cognition.Memory.Explicit | 2.3 |
|  |  | Cognition.Language.Phonology | 2 |  |  |  | Emotion.Positive | 2.94 |
|  |  | Cognition.Language.Semantics | 2.88 |  |  |  | Emotion.Valence | 3.49 |
|  |  | Cognition.Language | 3.36 |  |  |  | Cognition.Social Cognition | 3.57 |
|  |  | Cognition.Language.Syntax | 4.36 |  |  |  | Emotion.Negative.Sadness | 4.67 |
|  |  |  |  |  |  |  | Emotion.Negative.Anger | 4.79 |
|  | Area 46 | Cognition.Reasoning | 1.55 |  |  |  | Emotion.Positive.Happiness | 4.9 |
|  |  | Cognition.Memory.Working | 1.67 |  |  |  | Emotion.Negative.Disgust | 5 |
|  |  | Cognition.Language.Speech | 1.67 |  |  | Pir | Emotion.Negative | 2.48 |
|  |  | Cognition.Memory.Working | 2.04 |  |  |  | Emotion.Negative.Anger | 3.31 |
|  |  | Cognition.Language.Semantics | 2.15 |  |  |  | Interoception.Sexuality | 3.34 |
|  |  | Cognition.Language.Phonology | 2.47 |  |  |  | Emotion.Negative.Sadness | 4.43 |
|  |  |  |  |  |  |  | Emotion.Negative.Fear | 4.6 |
|  | p9-46v | Cognition.Language.Speech | 1.67 |  |  |  | Perception.Olfaction | 13.55 |
|  |  | Cognition.Memory.Working | 2.04 |  |  | TE1p | No significant effects |  |
|  |  | Cogntiion.Language.Semantics | 2.15 |  |  | PHT | Emotion.Negative.Disgust | 3.67 |
|  |  | Cognition.Language.Phonology | 2.47 |  |  | TPOJ2 | Action.Observation | 3.74 |
|  |  |  |  |  |  |  | Emotion.Negative.Disgust | 4.34 |
|  | a9-46v | Cognition.Memory.Working | 2.16 |  |  | TPOJ3 | Cognition.Social Cognition | 3.16 |
| Temporal |  |  |  |  |  |  | Perception.Vision.Shape | 3.67 |
|  | STSva | Cognition.Memory.Explicit | 1.98 |  |  |  | Cognition.Spatial | 4.62 |
|  |  | Cognition.Language.Semantics | 2.36 |  |  | TPOJ1 | Cognition.Social Cognition | 2.61 |
|  |  | Cognition.Social Cognition | 4.32 |  |  |  | Perception.Audition | 2.61 |
|  |  | Emotion.Valence | 5.34 |  | **Parietal** |  |  |  |
|  |  | Cognition.Language | 5.58 |  |  | PGi | Cognition.Social Cognition | 4.13 |
|  | A5 | Cognition.Music | 1.87 |  |  | PGs | Cognition.Social Cognition | 2.6 |
|  |  | Cognition.Language | 2.78 |  |  | IP1 | Cognition.Reasoning | 1.99 |
|  |  | Action.Execution.Speech | 2.96 |  |  |  | Cognition.Memory.Working | 2.41 |
|  |  | Cognition.Language.Speech | 3.31 |  |  | PFm | Cognition.Reasoning | 1.51 |
|  |  | Cognition.Language.Phonology | 3.76 |  |  |  | Perception.Somesthesis.Pain | 1.86 |
|  |  | Perception.Audition | 4.26 |  |  |  | Cognition.Memory.Working | 1.87 |
|  |  | Action.Motor Learning | 4.47 |  |  | LIPd | Cognition.Attention | 1.73 |
|  | STSdp | Cognition.Language.Semantics | 1.73 |  |  |  | Cognition.Reasoning | 1.9 |
|  |  | Cognition.Language.Speech | 2.11 |  |  |  | Perception.Vision | 1.98 |
|  |  | Cognition.Social Cognition | 2.15 |  |  |  | Action.Inhibition | 2.58 |
|  |  | Perception.Audition | 2.6 |  |  |  | Cognition.Spatial | 3.08 |
|  |  | Cognition.Language | 3.95 |  |  |  | Action.Observation | 3.78 |
|  | TE1m | Cognition.Language.Semantics | 2.31 |  |  |  | Perception.Vision.Color | 4.82 |
|  | TE1p | Cognition.Language.Speech | 2.34 |  |  | LIPv | Cognition.Attention | 1.48 |
|  |  | Cognition.Language.Orthography | 2.41 |  |  |  | Cognition.Memory.Working | 2.07 |
|  |  | Cognition.Language.Semantics | 2.48 |  |  |  | Perception.Vision.Shape | 2.96 |
|  |  | Cognition.Language.Phonology | 3.36 |  |  |  | Action.Observation | 3.38 |
|  | PHT | Cognition.Language.Semantics | 1.82 |  |  |  | Perception.Vision.Motion | 4.01 |
|  |  | Introception.Sexuality | 2.92 |  |  |  | Cognition.Spatial | 4.44 |
|  |  | Action.Observation | 4.46 |  |  | 7PC | Action.Execution | 1.88 |
|  | TPOJ1 | Cognition.Language.Semantics | 1.94 |  |  |  | Perception.Vision.Motion | 3.05 |
|  |  | Cognition.Language.Speech | 2.06 |  |  |  | Action.Motor Learning | 8.97 |
|  |  | Perception.Audition | 2.53 |  |  | 7AL | Action.Execution | 2.12 |
|  |  | Emotion.Valence | 3.84 |  |  |  | Action.Imagination | 4.49 |
|  | STV | Cognition.Social Cognition | 2.4 |  |  |  | Cognition.Spatial | 4.71 |
|  |  | Cognition.Music | 2.46 |  |  |  | Action.Motor Learning | 16 |
|  |  | Emotion.Valence | 3.96 |  |  | 7Am | Cognition.Memory.Working | 2.59 |
| Parietal |  |  |  |  |  |  | Action.Imagination | 3.5 |
|  | PFm | Cognition.Reasoning | 1.5 |  |  |  | Perception.Vision.Motion | 3.68 |
|  |  | Cognition.Memory.Working | 1.59 |  |  | 7Pm | Cognition.Memory.Working | 2.17 |
|  |  | Cognition.Social Cognition | 2.05 |  |  |  | Action.Inhibition | 2.77 |
|  | PGs | Cognition.Memory.Explicit | 1.88 |  |  |  | Perception.Vision.Motion | 3.06 |
|  | IP1 | Cognition.Reasoning | 1.81 |  |  | PCV | No significant effects |  |
|  | MIP | Cognition.Memory.Working | 1.79 |  |  | 5L | Emotion.Positive.Happiness | 9.19 |
|  |  | Cognition.Language.Orthography | 2.3 |  |  | 5mv | No significant effects |  |
|  |  | Perception.Vision.Shape | 2.45 |  |  | 23c | Emotion.Negative | 3.21 |
|  |  | Cognition.Spatial | 2.91 |  |  |  | Emotion.Positive.Happiness | 4.66 |
|  | LIPd | Perception.Vision | 1.87 |  |  | 3b | Action.Execution | 3.06 |
|  |  | Cognition.Language.Orthography | 2.54 |  |  |  | Perception.Gustation | 3.17 |
|  |  | Perception.Vision.Color | 3.55 |  |  |  | Action.Execution.Speech | 4.99 |
|  | VIP | Action.Execution | 2.54 |  |  |  |  |  |
|  |  | Perception.Vision.Motion | 3.96 |  |  |  |  |  |
|  | 7Am | Perception.Vision.Motion | 2.44 |  |  |  |  |  |
|  |  | Cognition.Spatial | 2.7 |  |  |  |  |  |
|  |  | Action.Imagination | 3.2 |  |  |  |  |  |
|  | 7Pl | Cognition.Attention | 1.64 |  |  |  |  |  |
|  |  | Cognition.Memory.Working | 2.04 |  |  |  |  |  |
|  |  | Perception.Vision.Shape | 2.63 |  |  |  |  |  |
|  |  | Cognition.Spatial | 2.98 |  |  |  |  |  |
|  |  | Perception.Vision.Color | 3.88 |  |  |  |  |  |
|  |  | Perception.Vision.Motion | 4.9 |  |  |  |  |  |
|  | 7Pm | Perception.Vision.Motion | 3.23 |  |  |  |  |  |
